# Residual Delta-Ferrite and Precipitated Phases in As-Cast 12.5% Ni 316L Stainless Steel: A Comparison Between Continuous Casting Billet and Directional Solidification

**DOI:** 10.3390/ma19173706

**Published:** 2026-08-31

**Authors:** Zhixuan Xue, Qi Zhao, Jiashuai Bian, Chaochao Pei, Dongzhi Hou, Lei Chen, Kun Yang, Zhou Li, Chao Chen

**Affiliations:** 1 College of Materials Science and Engineering, Taiyuan University of Technology, Taiyuan 030024, China; 2 Department of Mechanical Engineering, Taiyuan Institute of Technology, Taiyuan 030008, China; 3 College of Mechanical Engineering, Taiyuan University of Technology, Taiyuan 030024, China; 4 Institute of Materials Science, Technical University of Darmstadt, 64287 Darmstadt, Germany

**Keywords:** 12.5% Ni 316L austenitic stainless steel, ferrite morphology, ferrite content, secondary precipitated phases, solidification mode

## Abstract

The service performance of austenitic stainless steel is substantially affected by the presence of residual ferrite. In this paper, as-cast 12.5% Ni 316L austenitic stainless steel billets are taken as the research object, and samples are selected from the edge, quarter-thickness, and center positions of the billet, as well as two directionally solidified specimens prepared at different withdrawal speeds. By means of metallographic analysis, Thermo-Calc thermodynamic calculations, and EBSD phase analysis, the characteristics of residual ferrite and precipitated phases in the two types of as-cast 12.5% Ni 316L stainless steel were systematically investigated. The results show that the ferrite morphologies at the edge, quarter-thickness, and center positions of the billet are granular and short-rod, skeletal, and clustered net-like and lath-like, respectively. The ferrite morphologies of the two directionally solidified specimens are similar, both being predominantly skeletal structures; the main difference is that in the high-withdrawal-speed directionally solidified specimen (No. 2), the ferrite is finer and more densely distributed. The residual ferrite contents measured at the edge, quarter-thickness, and center positions of the billet are 4.88%, 5.90%, and 8.99%, respectively; those of directionally solidified specimens No. 1 and No. 2 are 6.4% and 7.7%, respectively. For the billet, the ferrite content increases progressively from the edge to the center. Regarding precipitated phases, the edge of the billet exhibits a mixed microstructure of secondary precipitates, namely Sigma phase and Chi phase; at the quarter-thickness position, the coupled precipitation of these two phases is more pronounced; at the center, part of the ferrite has completely decomposed, with the Chi phase disappearing and only the Sigma phase remaining. In the two directionally solidified specimens, only a small amount of the Sigma phase is precipitated as secondary phases, and the ferrite remains relatively intact. Based on the morphology analysis of the ferrite structure, the solidification mode of the billet is determined to be the FA mode, which is consistent with both the Scheil calculation results and the chromium-nickel equivalent calculation results; however, it differs from the thermodynamic equilibrium solidification results obtained using Thermo-Calc.

## 1. Introduction

Leveraging its exceptional corrosion resistance and high-temperature mechanical properties, austenitic stainless steel has found widespread application in critical industries such as petrochemicals, medical devices, and nuclear energy [1,2,3,4,5,6]. 316 austenitic stainless steel is based on 304 austenitic stainless steel with the addition of Ni and Mo elements [7,8,9,10]. 316L austenitic stainless steel traditionally has a standard Ni content ranging from 10 wt.% to 14 wt.% [11,12]. Under the Japanese Industrial Standard (JIS) designation SUS316L, the Ni content is above 12 wt.%. 316L austenitic stainless steel slabs produced via continuous casting typically retain a ferrite phase [13,14,15,16,17]. The presence of ferrite not only increases magnetic susceptibility, which affects its industrial applications [18,19,20,21,22], but also significantly impairs the corrosion resistance [23,24,25,26] and high-temperature stability [27,28,29,30] of the stainless steel. Furthermore, the precipitation of δ-ferrite is not exclusively observed in austenitic stainless steels; it can also occur in ferritic steels and duplex (ferritic–austenitic) cast steels [31]. Secondary precipitated phases, such as the Sigma phase, are commonly found in duplex stainless steels and are more stable [32], and they tend to precipitate in 316L stainless steel at high-temperature ranges. The presence of the Sigma phase also affects the properties of both 316L stainless steel and duplex stainless steels. Therefore, it is necessary to investigate the formation mechanisms of ferrite and precipitated phases in 316L austenitic stainless steel slabs. In this study, the term “residual delta ferrite” [13,33,34] specifically refers to the portion of δ-ferrite that precipitates during solidification and remains untransformed into austenite after the δ→γ diffusional transformation during the subsequent solid-state cooling stage, i.e., the ferrite that fails to completely convert to austenite.

The internal cooling rates within austenitic stainless steel slabs vary considerably [35,36,37,38], leading to differences in ferrite morphology, content, and phase precipitation at different positions across the slab [39,40,41,42,43]. The non-uniform distribution of δ-ferrite in austenitic stainless steel slabs was first reported by Lindenberg et al. [44] in 1984. Subsequently, Wolf et al. [45] demonstrated that the ferrite distribution in 304 stainless steel slabs also exhibits variations. In 1995, Kim et al. [33] further found that the ferrite content in 304 austenitic stainless steel slabs differs along the thickness direction: approximately 4% at the surface, rising to a maximum of 9% at 95 mm from the surface, and then decreasing to 6% at the slab center. Chen and Cheng [46] summarized the ferrite distribution patterns in slabs, generally presenting M-type and A-type distributions. For 316H slabs, it was reported by Wang et al. [47] that the cooling rate drops from 1.3 °C/s at the casting surface to 0.2 °C/s at the center. Along with this change, only a small amount (<3%) of cellular δ-ferrite is observed in the surface region, whereas the central region exhibits up to 8% of skeletal δ-ferrite. Li et al. [48] reported the ferrite distribution characteristics of 316H heavy plates with a Ni content of 12 wt.%, finding that the ferrite content increases from the slab edge to the center, reaching up to 15% at the center. For 316L billets, Pei et al. [49] studied 316L billets with 10 wt.% Ni and found that from the surface to 85 mm along the width direction, the ferrite area fraction increases from 4.2% to 10.6%, then decreases toward the center to 7.8%, with the ferrite morphology sequentially changing from granular and skeletal → network → lath → short rod-like. Several studies have addressed precipitation behavior in austenitic stainless steels. Wang et al. [50] analyzed 316H ESR ingots and identified both Chi and Sigma phases at the edge and center. Separately, Chu et al. [51] focused on 316L slabs with 11.38% Ni, observing that the Sigma phase is rare at the surface but increases in the quarter-thickness and central areas, where more ferrite transforms into the Sigma phase.

The solidification modes of austenitic stainless steel can be classified into four types: A, AF, FA, and F. Among these, the AF and FA modes correspond to the processes in which austenite or ferrite nucleates and grows as the primary precipitated phase during the initial stage of solidification, respectively. The composition of 316 stainless steel lies near the peritectic point [52], exhibiting both FA and AF solidification modes [53,54]. Li et al. [55] investigated the solidification process of 316H austenitic stainless steel over a cooling rate range of 5 °C/min to 6000 °C/min and determined that the solidification mode is consistently of the FA type. Wang et al. [56] conducted real-time observations of the solidification process of 316L samples with varying nickel contents using high-temperature confocal laser scanning microscopy (HT-CLSM), finding that low-nickel 316L steel solidifies in the FA mode, whereas high-nickel 316L steel solidifies in the AF mode. Recent studies [57,58] have reported that 316L stainless steel with 12.17% nickel adopts the FA solidification mode. For 316L austenitic stainless steel with a nickel content of 12.5%, the solidification mode may be either AF or FA. To date, there has been limited research on the solidification mode of 316L austenitic stainless steel with 12.5% nickel content.

In the present work, specimens were prepared by billet sampling and directional solidification experiments. Metallographic analysis, Thermo-Calc thermodynamic calculations, and electron backscatter diffraction (EBSD) phase analysis were employed to systematically investigate the characteristics of residual ferrite and precipitated phases in two as-cast 12.5% Ni 316L stainless steels.

## 2. Materials and Methods

A 316L austenitic stainless steel billet produced by continuous casting at a certain plant was selected, with a billet size of 180 mm × 180 mm. The main chemical components determined by inductively coupled plasma optical emission spectrometry (ICP) are shown in Table 1. The Ni content is 12.57%, which simultaneously meets the requirements of the traditional standard (10.00–14.00%) [11,12] and the Japanese standard (12.00–15.00%). The Cr content is 17.60%, falling in the upper middle of the specified range of 16.00–18.00% and thus complying with the standard. The Mo content is 2.53%, within the specified range of 2.00–3.00%. The contents of C, Si, Mn, P, S, and N are all below the upper limits specified by the standards; in particular, the C content (0.014%) is far below the upper limit of 0.030%, and the S content (0.0017%) is also far below the upper limit of 0.030%, indicating a high level of material cleanliness. The designation “12.5% Ni 316L stainless steel” is a concise expression based on the measured Ni content of 12.57%, intended to distinguish 316L stainless steels with different Ni content levels; it does not refer to any specific non-standard grade.

Using medium-nickel 316L stainless steel billets as the base material, directional solidification experiments were conducted in a directional solidification furnace. A high-purity argon protective atmosphere was employed to prevent specimen oxidation, and a water-cooling mechanism was applied to maintain forced cooling at the bottom of the specimens, thereby establishing a stable temperature gradient. The temperature gradient was set at 20 K/mm, and two directional solidification specimen strips were prepared at withdrawal rates of 100 μm/s and 400 μm/s, respectively. The designed combination of casting speed and temperature gradient corresponds to the cooling rates at different positions of the billet. A casting speed of 400 μm/s corresponds to a cooling rate of approximately 8.0 °C/s, which is close to the cooling rate at the billet edge. In addition, a scheme with a lower cooling rate was selected, where a casting speed of 100 μm/s corresponds to a cooling rate of about 2.0 °C/s. The specimen strips had dimensions of 90 mm × 10 mm × 10 mm, and were denoted as Directional Solidification Specimen 1 (DS 1) and Directional Solidification Specimen 2 (DS 2).

**Figure 1:**
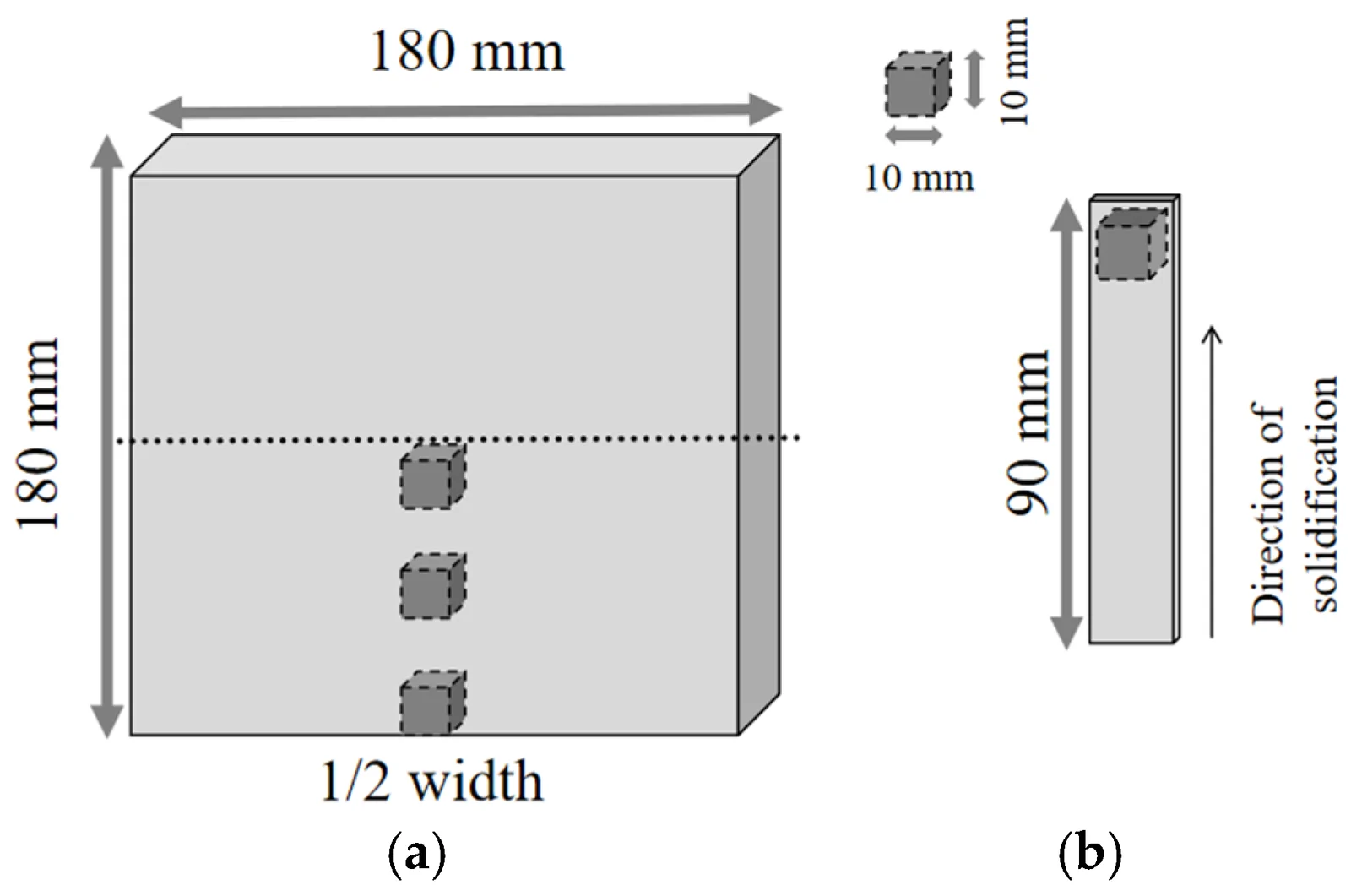
Schematic diagram of sampling. (**a**) 316L billet; (**b**) directional solidification specimen rod.

The sampling scheme is illustrated in Figure 1. One specimen was taken from each of the edge, quarter-section, and center positions of the 316L stainless steel billet (previous studies [51,59] have also focused on the evolution of ferrite morphology, content, and secondary phase precipitation behavior along this direction from the billet edge to the center; thus, selecting these three typical positions can comprehensively reflect the differences in the as-cast microstructure). One specimen was taken from the top of each of the two directionally solidified specimen bars (the final solidification positions, where the morphology of retained ferrite and the evolution of precipitated phases have undergone long-term changes, making the conditions relatively similar to those at the quarter-thickness and center positions of the slab, which is beneficial for observing the final retained state of ferrite and the precipitation of secondary phases); a total of five specimens were obtained, each with dimensions of 10 mm × 10 mm × 10 mm.

The obtained specimens were subjected to grinding, polishing, and etching (in a mixed solution of 0.5 g FeCl_3_, 5 mL HCl, and 10 mL H_2_O). The ferrite content was then determined by a metallographic method [60] using Image-Pro-Plus (IPP, version: Image-Pro-Plus 7.0) software. At least 10 fields of view on the specimen surface were observed under a ZEISS AXIO optical microscope at 100× magnification. A manual threshold segmentation method was adopted: the threshold separating the austenite matrix (gray) from the ferrite phase (black) was selected based on the gray-level histogram. The threshold setting was performed by the same operator under identical illumination conditions to ensure consistency. The ferrite content (volume fraction) in each field of view was measured using the IPP software, and the average value was calculated. Subsequently, the specimens were electropolished (in a 10 vol% HClO_4_ ethanol solution) to prepare samples for electron backscatter diffraction (EBSD) analysis, in order to obtain the crystal orientation and phase precipitation information of each specimen. The specimen preparation method was consistent with that reported in previous studies [57,58]. In addition, the high-magnification morphology of ferrite was observed using scanning electron microscopy (SEM), and the elemental composition of the microstructure was analyzed by both area mapping and point scanning using the attached energy-dispersive X-ray spectroscopy (EDS).

Based on the composition given in Table 1, the phase evolution during equilibrium solidification of the 316L stainless steel was calculated using the Thermo-Calc thermodynamic software (version: Thermo-Calc 2025a) with the TCFE13 database.

## 3. Results

### 3.1. Experimental Results of the 316L Stainless Steel Billet

#### 3.1.1. Metallographic Observation Results of the Billet

**Figure 2:**
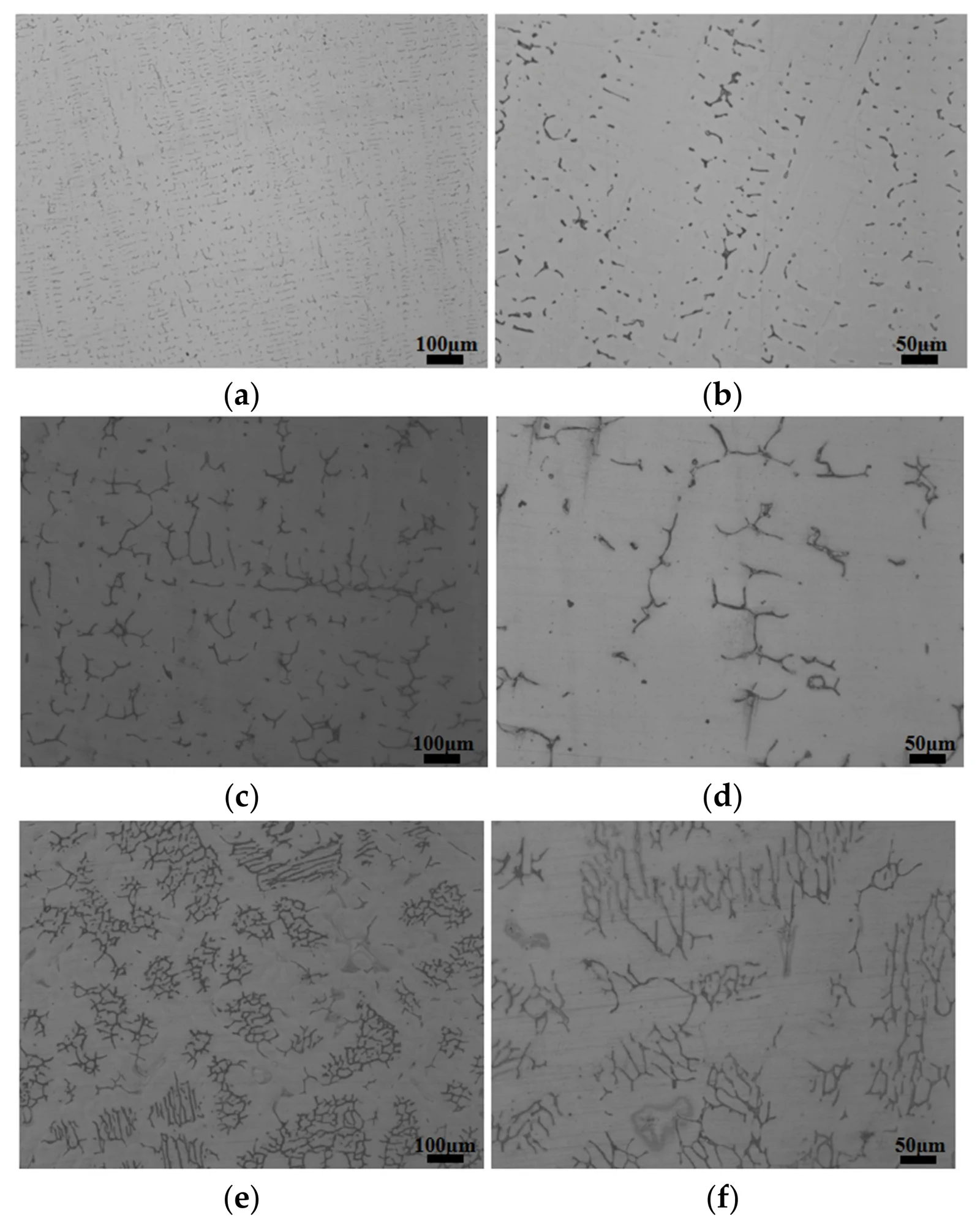
Metallographic observation results of the 316L stainless steel billet. (**a**,**b**) billet edge; (**c**,**d**) quarter-thickness position; (**e**,**f**) billet center.

Figure 2 shows the metallographic observation results of the edge, quarter-thickness, and center positions of the 316L austenitic stainless steel billet. The left column (a) (c) (e) shows images at 100× magnification, while the right column (b) (d) (f) shows images at 200× magnification. The gray matrix is the austenite phase, and the black constituent is the ferrite phase. It can be seen from Figure 2 that the ferrite morphology differs significantly at different positions of the 316L stainless steel billet. At the billet edge, the ferrite morphology is mainly granular and parallel short rod-like structures, and the ferrite dendrites are fine and relatively uniformly distributed. Unlike at the edge, at the quarter-thickness position, the ferrite morphology is more interconnected into complex skeletal structures, and in Figure 2d, some skeletal ferrite regions exhibit non-uniform internal distribution. Skeletal ferrite is typically a product of the FA solidification mode [61]. At the billet center, the ferrite morphology is even more distinctive, with ferrite distributed in clustered regions approximately 100 μm in diameter; the morphology mainly consists of clustered networks and lath-like structures, and the ferrite dendrites are noticeably coarser.

#### 3.1.2. Cooling Rate and Ferrite Content of the Billet

Currently, researchers often use the secondary dendrite arm spacing (SDAS) in the solidified structure to indirectly estimate the cooling rate [47], primarily via Equation (1) [62,63].

λ_2_ = 44ν^−0.38^ (1)

where λ_2_ is the SDAS (unit: μm); ν is the cooling rate (unit: °C/s).

**Figure 3:**
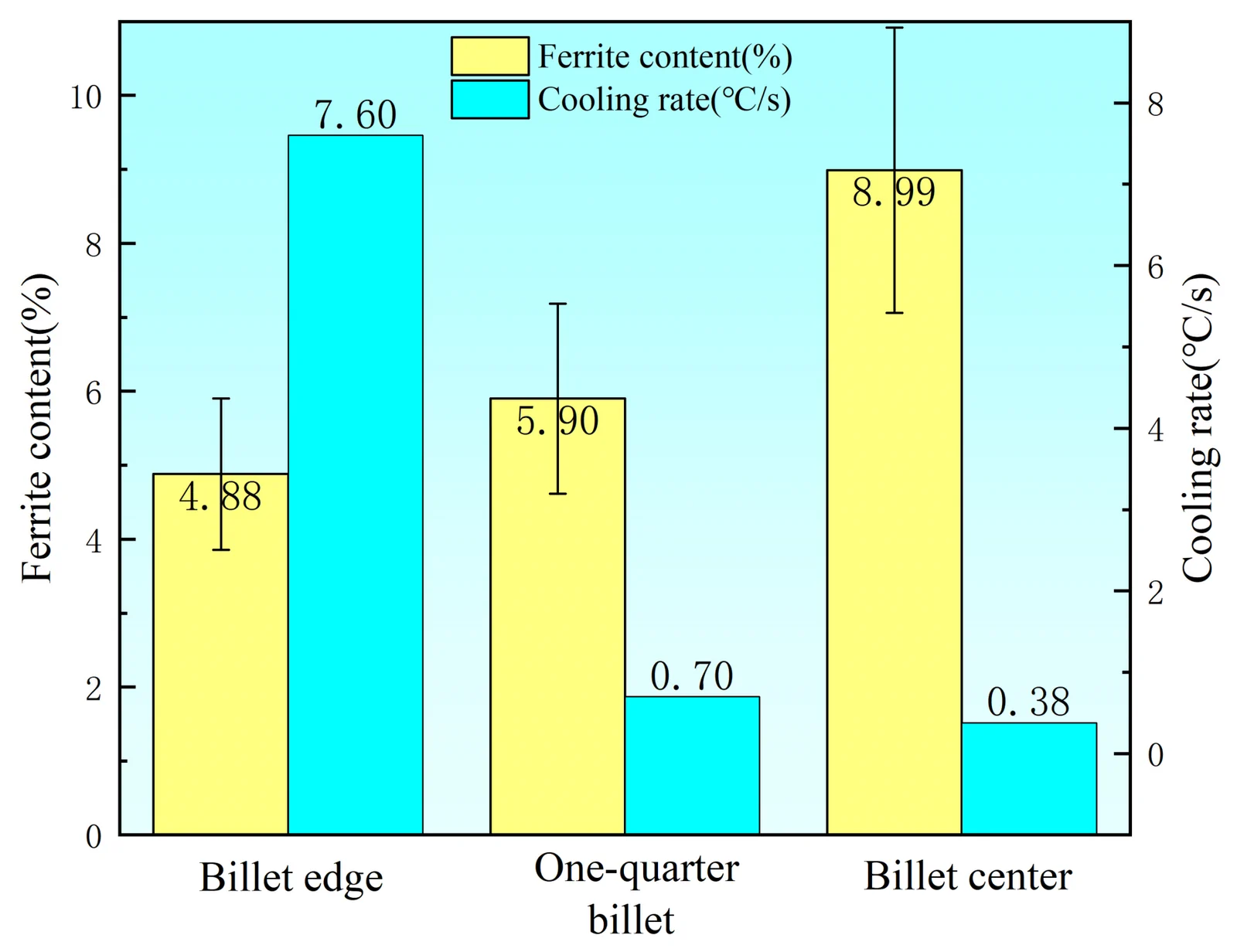
Ferrite content and cooling rate at different positions of the 316L stainless steel billet.

Shown in Figure 3 are the ferrite content and cooling rate results at different positions of the 316L billet. It can be seen that the ferrite content tends to increase from the billet edge to the center; the ferrite contents at the edge, quarter-thickness, and center positions are 4.88%, 5.90%, and 8.99%, respectively. The corresponding cooling rates at these positions are calculated to be 7.6 °C/s, 0.70 °C/s, and 0.38 °C/s, respectively. Therefore, the cooling rate gradually decreases from the billet edge to the center, and there is a significant difference in cooling rate between the edge and center positions.

#### 3.1.3. Secondary Precipitated Phases in the Billet

**Figure 4:**
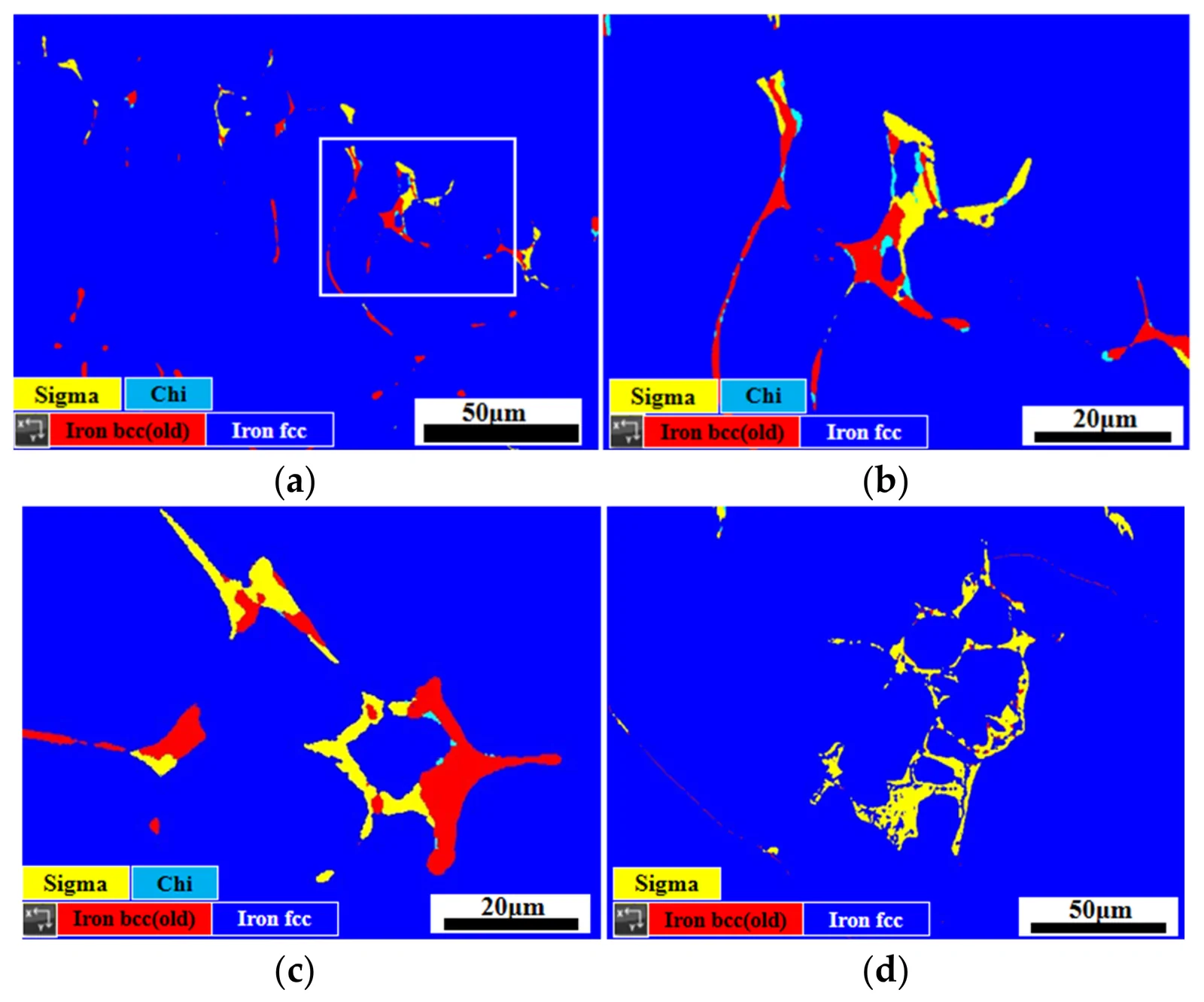
EBSD of 316L stainless steel billet. (**a**) Edge of the billet; (**b**) high-magnification image of precipitates at the edge; (**c**) quarter section of the billet; (**d**) center of the billet.

The 316L stainless steel billet specimen analyzed by EBSD is shown in Figure 4. From Figure 4a, it can be seen that the phase distribution at the edge of the billet is relatively complex; in addition to the austenite matrix and ferrite, there are also the Sigma phase and a small amount of the Chi phase. A higher-magnification examination was performed on the white-boxed area in Figure 4a, as shown in Figure 4b, which clearly reveals a coupled structure of ferrite, Sigma, and Chi phases at the edge. A similar coupled structure of ferrite, Sigma, and Chi phases also appears at the quarter-section position. However, the secondary precipitate phase at the center of the billet was found to consist solely of the Sigma phase, with the Chi phase disappearing, and it is evident from Figure 4d that ferrite has almost completely decomposed and transformed into the Sigma phase. From Figure 4, it can be seen that the secondary precipitates precipitate both within the ferrite and at the ferrite/austenite phase boundaries. The EBSD results indicate that the contents of Sigma and Chi phases at the edge are approximately 0.6% and 0.063%, respectively; at the quarter-section, they are approximately 2.85% and 0.085%, respectively; and at the center, the Sigma phase content is approximately 3.01%.

**Figure 5:**
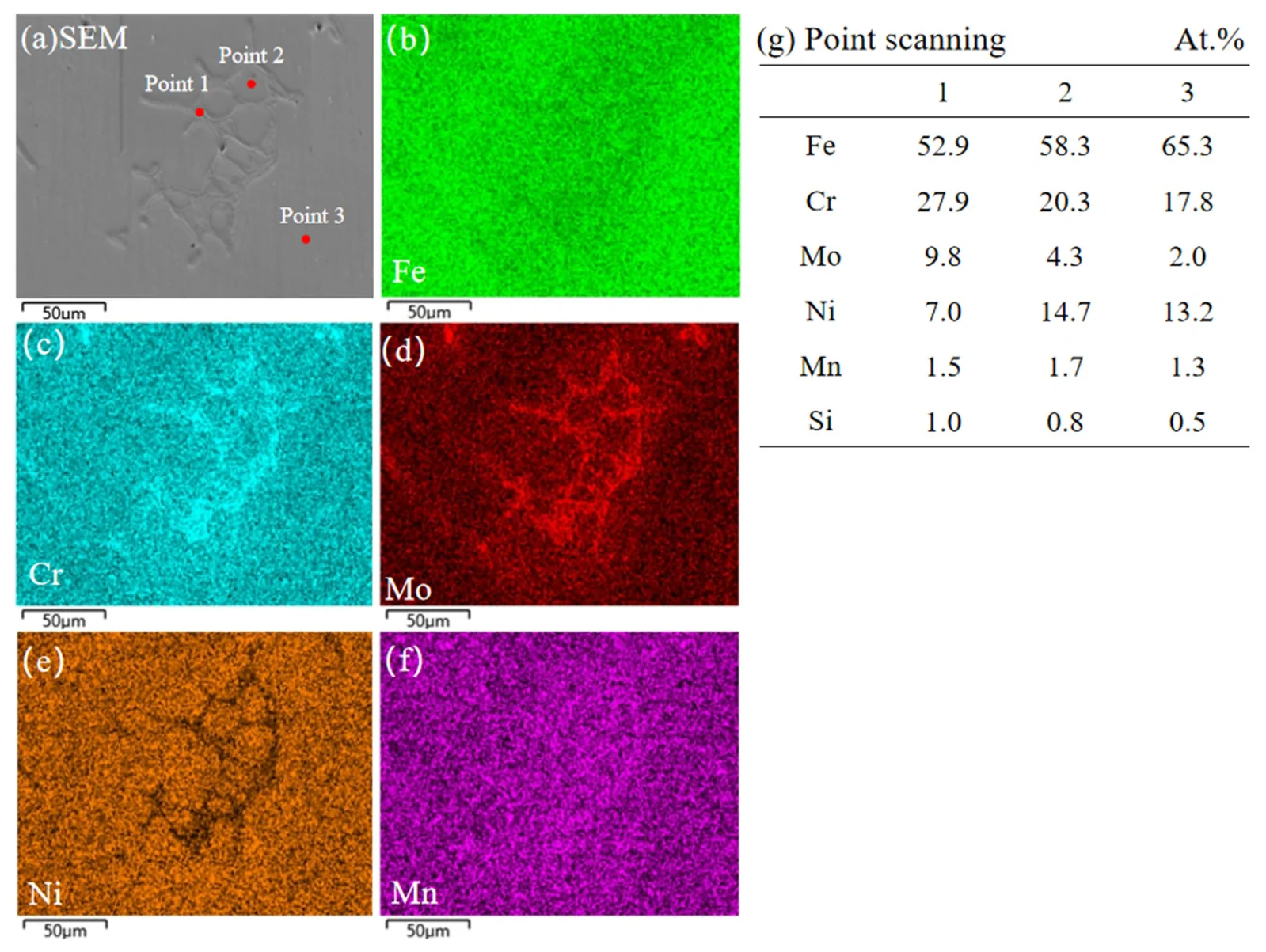
Elemental analysis results of the 316L billet center sample. (**a**–**f**) area scanning; (**g**) point scanning.

SEM-EDS analysis was performed on the precipitated phases in the billet center sample shown in Figure 4d, and the results are presented in Figure 5. From the results in Figure 5a–f, it can be seen that the Sigma phase is severely enriched in Cr and Mo elements and depleted in Ni. Point scanning measurements in Figure 5g at points 1, 2, and 3 correspond to the secondary precipitated Sigma phase, the austenite surrounded by Sigma, and the austenite matrix outside the Sigma phase, respectively. The results show that at point 1 on the secondary precipitated Sigma phase, the Cr content is 27.9%, Mo is 9.8%, and Ni is 7.0%; the Cr and Mo contents at point 1 are much higher than those at points 2 and 3, while the Ni content is much lower than that at points 2 and 3. Points 2 and 3 are both in austenite, but the elemental contents of Cr, Mo, and Ni at point 2 (20.3%, 4.3%, 14.7%) are all higher than those at point 3 (17.8%, 2.0%, 13.2%).

### 3.2. Experimental Results of Directional Solidification of 316L Stainless Steel

**Figure 6:**
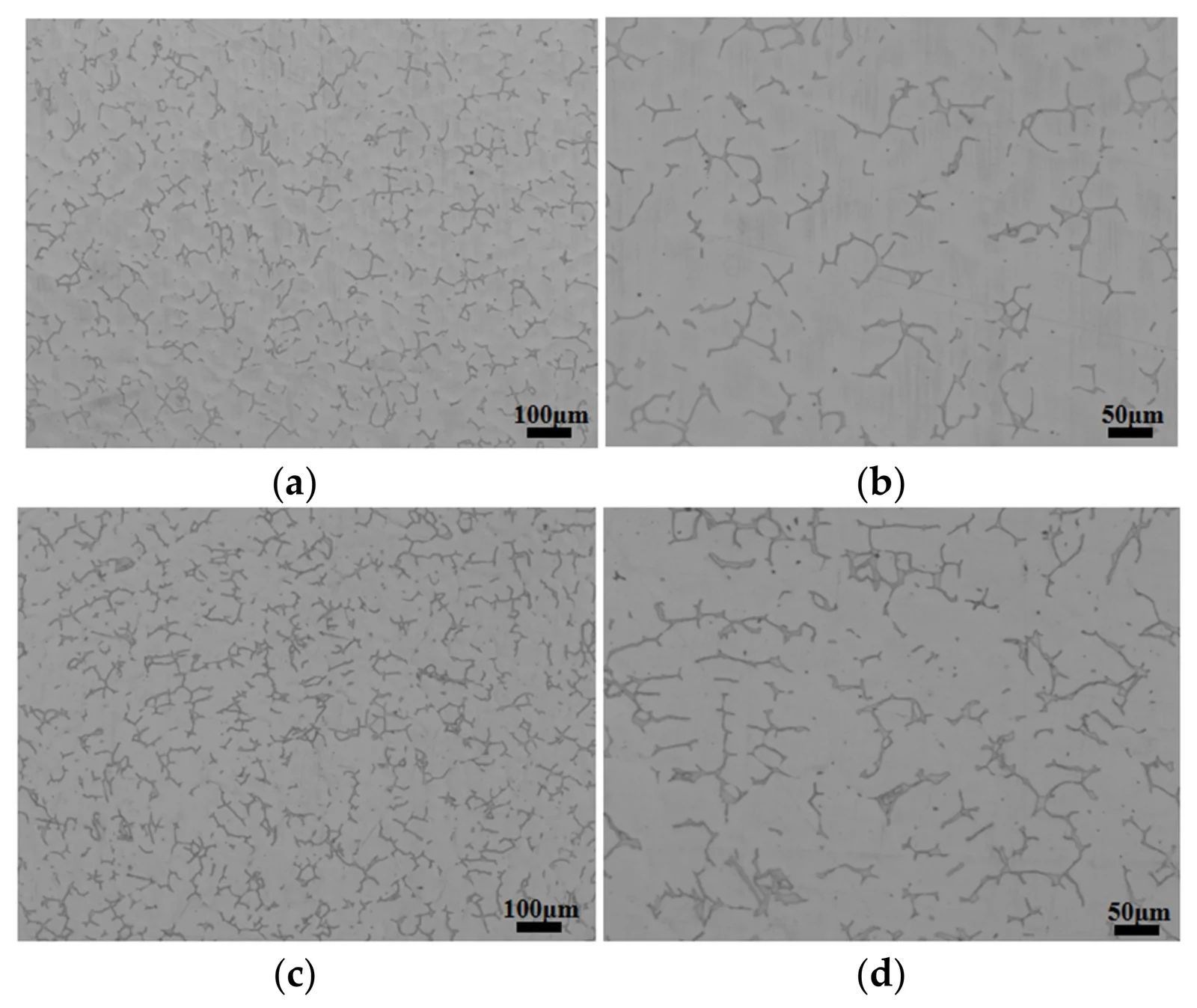
Metallographic observation results of directional solidification specimens of 316L stainless steel. (**a**,**b**) DS 1; (**c**,**d**) DS 2.

Shown in Figure 6 are the metallographic observation results of DS 1 and DS 2 of the 316L stainless steel. The left column (a) (c) shows images at 100× magnification, and the right column (b) (d) at 200× magnification. It can be seen that the ferrite morphologies of the two directional solidification specimens are similar, both mainly exhibiting a skeletal structure, and the ferrite is uniformly distributed. Compared with the billet, the directional solidification specimens show a more uniform and regular microstructure, and the skeletal ferrite tends to grow in the same direction. The main difference in ferrite between the two directional solidification specimens with different withdrawal rates is that in Specimen 2, which was produced at a higher withdrawal rate, the ferrite is denser and has a higher volume fraction.

**Figure 7:**
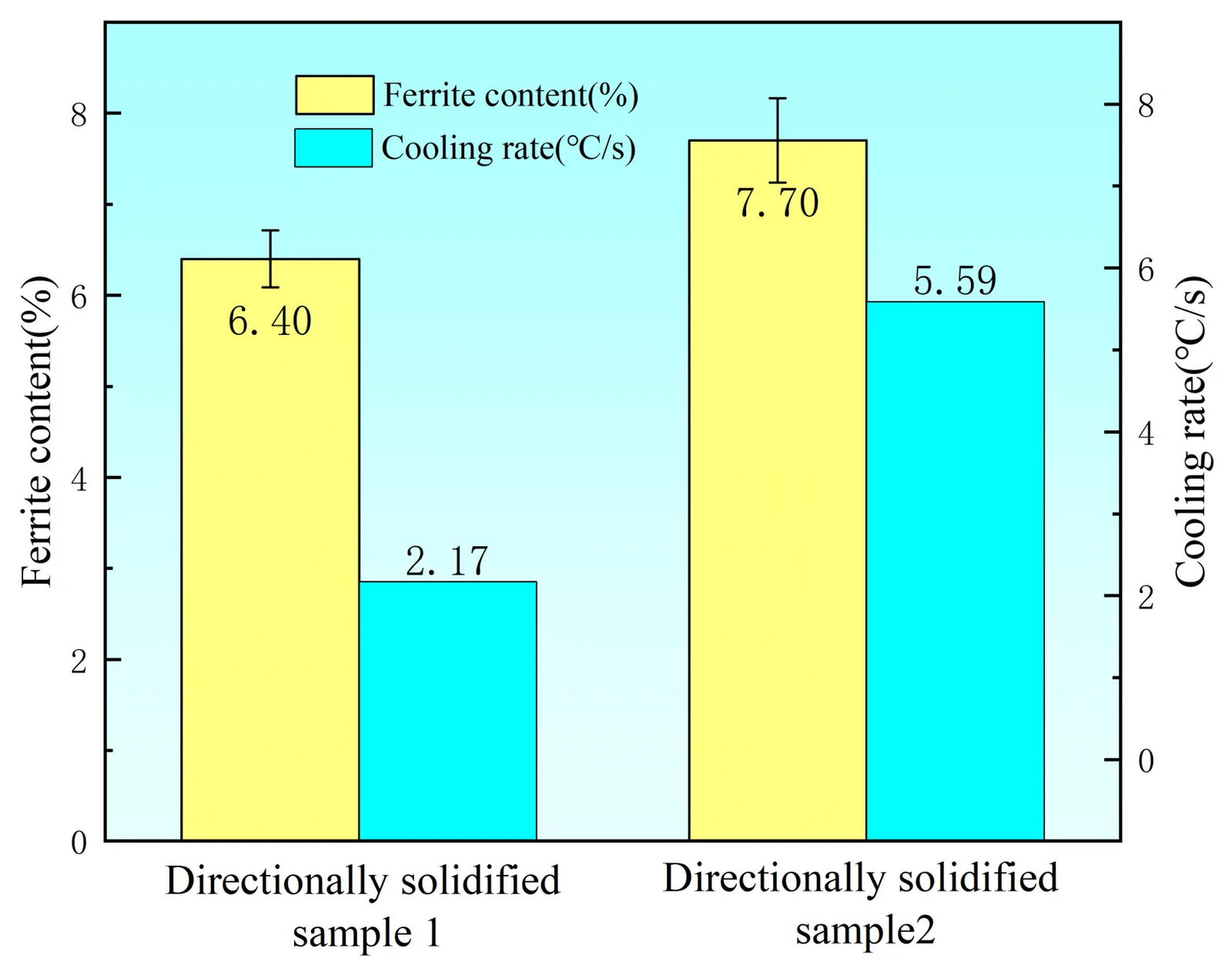
Ferrite content and cooling rate of directional solidification specimens.

As shown in Figure 7, the ferrite contents of DS 1 and DS 2 are 6.4% and 7.7%, respectively, and the corresponding cooling rates calculated according to Equation (1) are 2.17 °C/s and 5.59 °C/s, respectively. The directional solidification specimens demonstrate that the ferrite content of the specimen with a higher cooling rate is greater than that of the specimen with a lower cooling rate.

**Figure 8:**
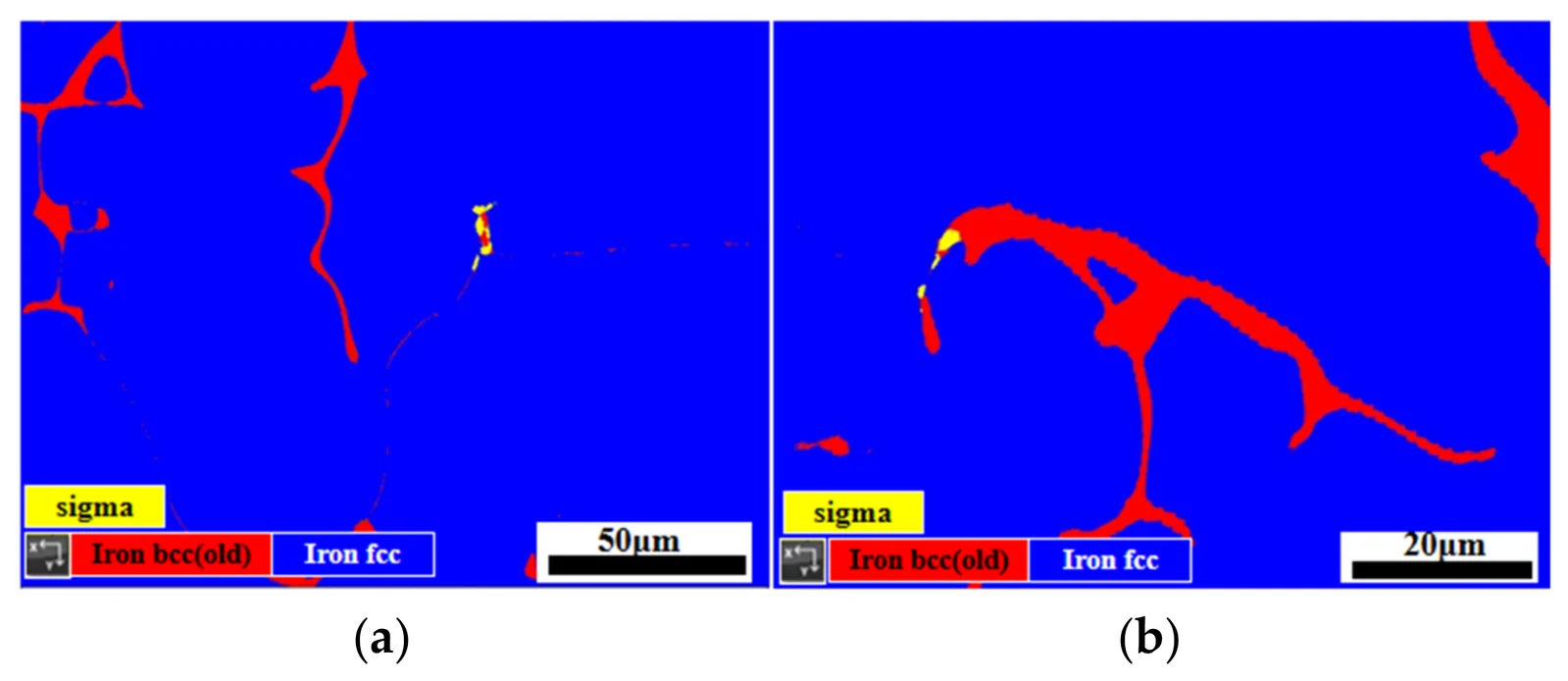
EBSD of directional solidification specimens of 316L stainless steel. (**a**) DS 1; (**b**) DS 2.

Figure 8 shows the EBSD of the two directional solidification specimens of 316L stainless steel. From the results, it can be seen that in Directional Solidification Specimen 1, most of the ferrite remains intact, and only a small patch of ferrite at the ferrite/austenite phase boundaries has been relatively completely transformed into the Sigma phase. In Directional Solidification Specimen 2, a small region on the ferrite dendrite arms has transformed into the Sigma phase. Both Directional Solidification Specimens 1 and 2 show partial transformation of ferrite into the Sigma phase, but the majority of the ferrite has not decomposed and remains relatively intact. EBSD results show that the Sigma phase contents of the secondary precipitates in Directional Solidification Specimens 1 and 2 are approximately 0.1% and 0.098%.

### 3.3. Thermo-Calc Thermodynamic Calculation Results

**Figure 9:**
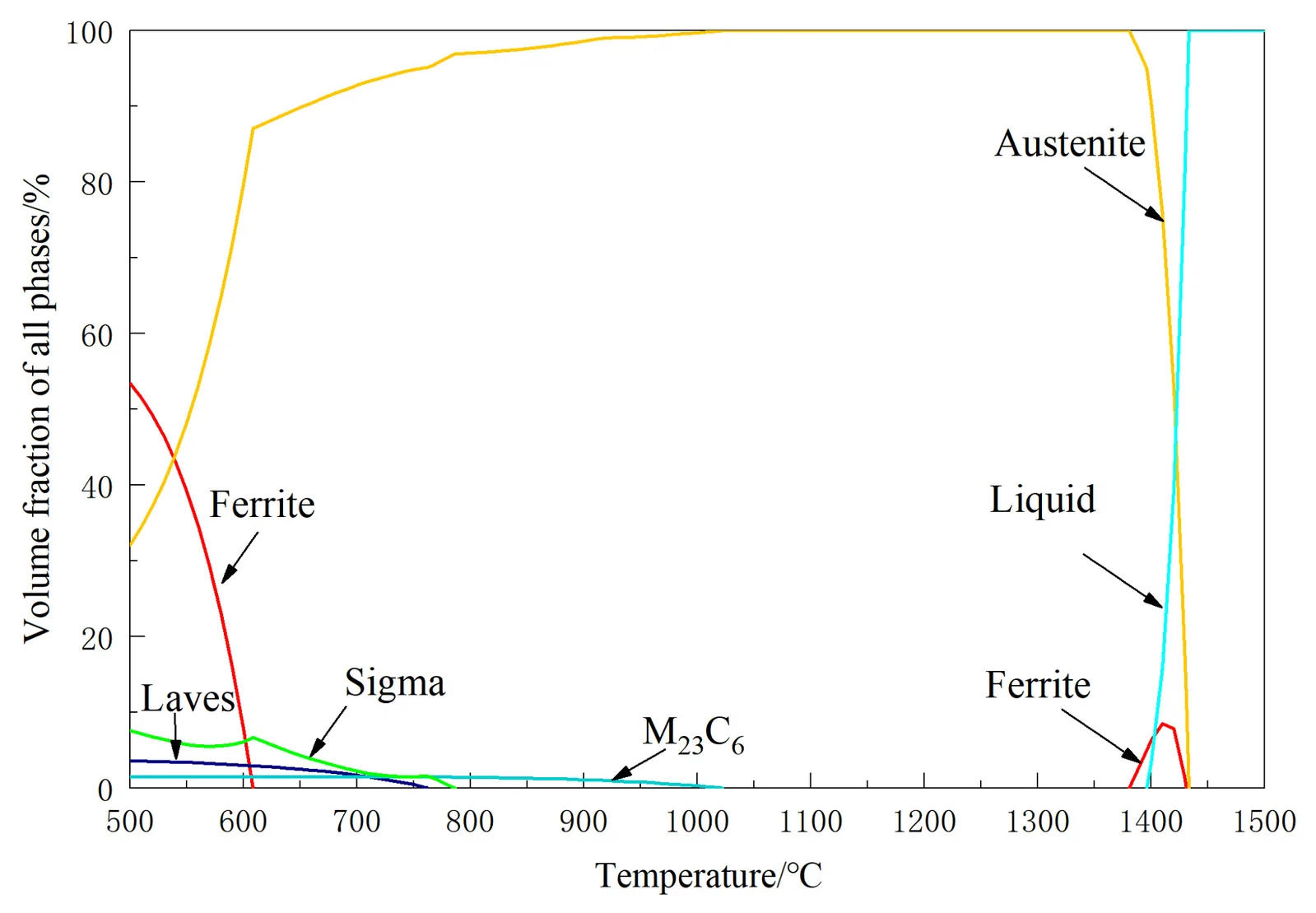
Equilibrium solidification process of the 316L billet.

Based on the composition given in Table 1, the phase evolution during solidification of 316L austenitic stainless steel from 1500 °C to 500 °C was calculated using Thermo-Calc software, and the results are shown in Figure 9. It can be seen from Figure 9 that the main solidification austenite phase precipitates from the liquid phase at 1433 °C, followed by ferrite precipitation at 1431 °C. The peak volume fraction of ferrite in the high-temperature region is 8.5%. When the temperature decreases to 1395 °C, the liquid phase disappears, and then a solid-state phase transformation between ferrite and austenite occurs. At 1380 °C, the ferrite disappears, and upon further cooling, the alloy enters the fully austenitic phase region. The Sigma phase appears at 786 °C, and α-ferrite appears at 608 °C. The temperature range of 1395–1431 °C is a three-phase region where L + δ + γ coexist, and 1380–1395 °C is a two-phase region where δ + γ coexist. From Figure 9, the solidification sequence can be described as L → L + γ → L + δ + γ → δ + γ → γ → γ + α + precipitates. The precipitation temperature of austenite and that of ferrite are extremely close, differing by only 2 °C, and the equilibrium solidification result is a weak AF mode.

**Figure 10:**
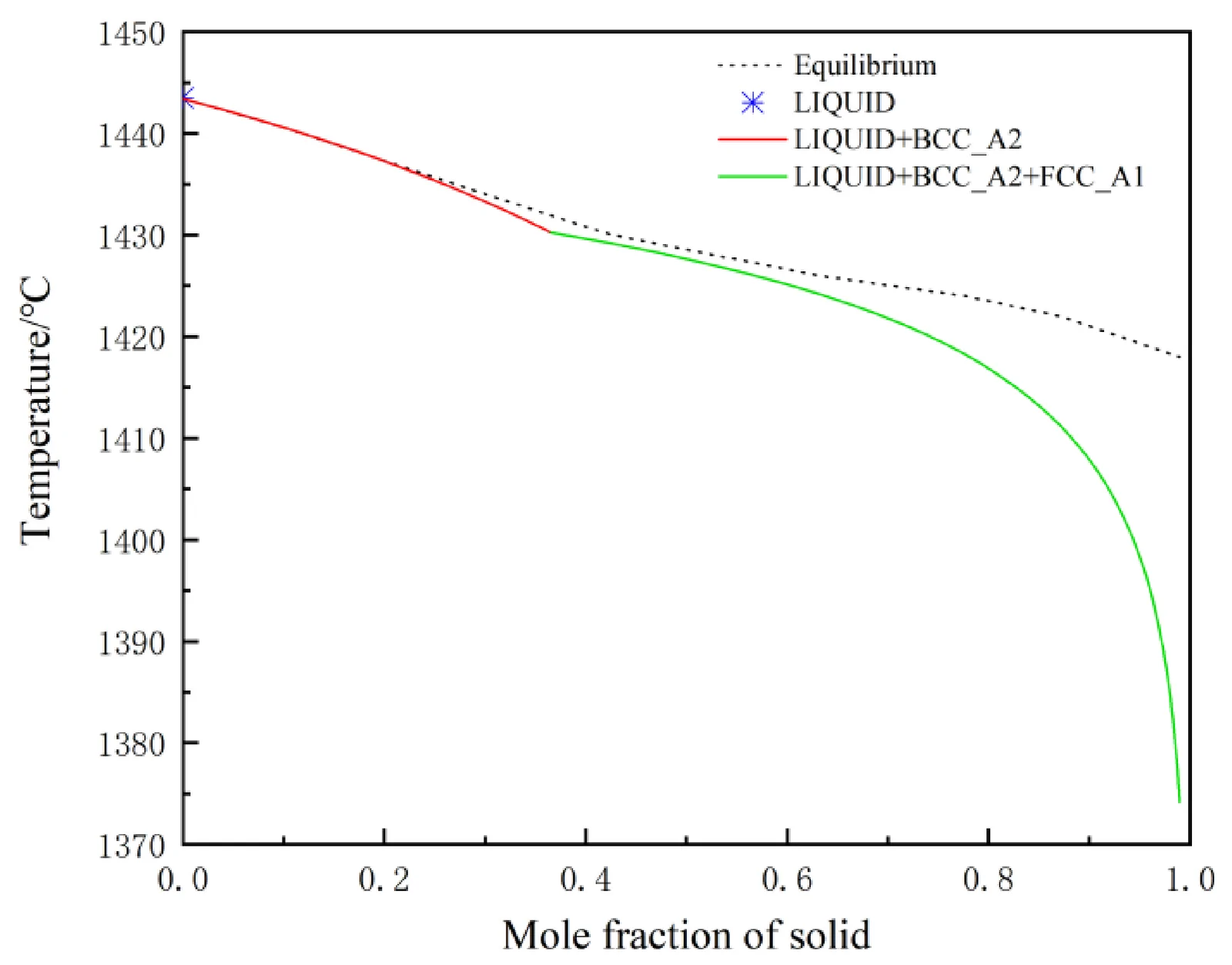
Non-equilibrium solidification process of the 316L billet calculated by the Scheil model.

Figure 10 shows the solidification process of the 316L billet under non-equilibrium conditions, calculated using the Scheil model in Thermo-Calc. The Scheil model is an ideal non-equilibrium solidification model, which assumes complete diffusion of elements in the liquid phase and no diffusion in the solid phase. The results show that in the 316L billet, ferrite begins to precipitate as the primary phase, with ferrite precipitating at 1443 °C and austenite at 1430 °C. Ferrite completely disappears at 1374 °C, at which point the volume fraction of austenite is 98.8%, which is close to the equilibrium thermodynamic calculation results. It can be concluded that the non-equilibrium solidification mode of the 316L billet is the FA mode.

## 4. Discussion

### 4.1. Solidification Mode of 316L Stainless Steel

The solidification mode of austenitic stainless steel is mainly determined by its composition, and the cooling rate also has a certain influence on the solidification mode. The composition of austenitic stainless steel is relatively complex; it can be simplified into the Fe–Cr–Ni ternary system based on the chromium equivalent (Cr_eq_) and nickel equivalent (Ni_eq_). The solidification modes can be classified into the following four types:

A mode: L → L + γ → γ  (Cr_eq_/Ni_eq_) < 1.25

AF mode: L → L + γ → L + γ + δ → γ + δ  1.25 < (Cr_eq_/Ni_eq_) < 1.48

FA mode: L → δ + γ → L + δ + γ → δ + γ  1.48 < (Cr_eq_/Ni_eq_) < 1.95

F mode: L → L + δ → δ → δ + γ  1.95 < (Cr_eq_/Ni_eq_)

Among them, L represents the liquid phase; δ represents ferrite; γ represents austenite. The solidification mode of austenitic stainless steel is usually determined by the empirical Cr_eq_/Ni_eq_ ratio formula. Equations (2) and (3) are the formulas proposed by Hammar-Svensson [64], which are currently among the most widely used:

Cr_eq_ = ω(Cr) + 1.37 × ω(Mo) + 1.5 × ω(Si) + 0.5 × ω(Nb) (2)

Ni_eq_ = ω(Ni) + 22 × ω(C) + 0.31 × ω(Mn) + 14.2 × ω(N) + ω(Cu) (3)

Using the above formulas, the Cr_eq_ value for the 12.5% Ni 316L austenitic stainless steel is calculated to be 21.83, the Ni_eq_ value is 14.01, and the Cr_eq_/Ni_eq_ ratio is 1.56. When 1.48 < (Cr_eq_/Ni_eq_) < 1.9, the solidification mode is classified as FA. Therefore, the empirical chromium-nickel equivalent formula predicts that the solidification mode of the 316L stainless steel is the FA mode.

From the results shown in Figure 9 and Figure 10, it can be seen that the equilibrium and non-equilibrium solidification processes of the 316L stainless steel calculated by Thermo-Calc yield AF and FA modes, respectively. The predictions obtained using the chromium-nickel equivalent empirical formula are consistent with those derived from the non-equilibrium solidification process, both yielding the FA mode; in contrast, the solidification mode predicted by the Thermo-Calc equilibrium calculation is the AF mode. It is well known that under the FA solidification mode, ferrite morphologies typically include skeletal ferrite, lath-like ferrite, blocky ferrite, etc. [61]; under the AF mode, ferrite is usually distributed along austenite grain boundaries, nucleating in the remaining liquid phase after the formation of austenite [56]. In the 316L billet samples from the edge, quarter-thickness, and center positions, the ferrite morphologies are mostly skeletal and lath-like structures; the directional solidification specimens also exhibit predominantly skeletal ferrite. In none of the five samples was ferrite observed to be distributed solely along austenite grain boundaries. Moreover, the actual solidification process of stainless steels is mostly a non-equilibrium process. Considering all the above evidence, it can be concluded that the solidification mode of the 316L stainless steel is the FA mode.

### 4.2. Ferrite Characteristics of the Billet and Directional Solidification Specimens

Table 2 summarizes the ferrite content, cooling rate, and precipitation behavior of the 316L stainless steel billet and directional solidification specimens. From Table 2, it is evident that the evolution of ferrite content and secondary precipitates varies slightly among samples from different sources.

Based on the results summarized in Table 2, the residual ferrite in the 316L stainless steel billet and the directional solidification specimens exhibits different trends. In the billet, the cooling rate is highest at the edge (7.60 °C/s), where the ferrite morphology is fine short rod-like and granular, and the ferrite content is lowest (4.88%). At the center, the cooling rate is lowest (0.38 °C/s), where the ferrite grows into complex clustered networks and the ferrite content is highest (8.99%). At the quarter-thickness position, the ferrite is skeletal, with a ferrite content (5.90%) and cooling rate (0.70 °C/s) intermediate between those of the edge and center. Thus, within the billet, the ferrite content increases from the edge to the center as the cooling rate decreases. The directional solidification results show a different behavior: both directional solidification specimens exhibit skeletal ferrite morphology. DS 2, with a higher cooling rate of 5.59 °C/s, has a ferrite content of 7.70%; while specimen 1, with a lower cooling rate of 2.17 °C/s, has a ferrite content of 6.40%. At higher cooling rates, the ferrite transformation is incomplete, whereas at lower cooling rates, sufficient diffusion allows more ferrite to be consumed. The cooling rates of the directional solidification specimens lie between those of the billet edge and center, and their ferrite contents also fall in between.

From the billet edge to the center, the ferrite morphology of the 316L stainless steel sequentially changes from granular and short rod-like to skeletal, and then to clustered networks and lath-like structures, with the ferrite content increasing from 4.88% to 8.99%. These morphology and content results differ from those reported by Pei et al. [49] for low-Ni (10.1% Ni) 316L austenitic stainless steel billets. In Pei et al.’s study, the ferrite morphology from the edge to the center changed sequentially from granular and skeletal → network → lath → short rod-like, with a ferrite content ranging from 4.2% to 10.6%, exhibiting an “M-shaped” distribution (first increasing and then decreasing). Wang et al. [50] studied a 316H electroslag remelted ingot with a Ni content (12.2%) similar to that of the present study (12.5% Ni). In their work, the ferrite morphology from the edge to the center was granular, short rod-like, blocky, skeletal, and network, which is similar to the morphology changes observed in the present 12.5% Ni 316L billet. However, their ferrite content only ranged from 1.92% to 3.57%, which is much lower than that found in the present study.

### 4.3. Characteristics of Secondary Precipitated Phases in Billet and Directionally Solidified Specimens

Within the 316L billet, from the edge to the center, the secondary precipitates successively are Sigma and Chi phases, Sigma and Chi phases, and finally only the Sigma phase. In the two directional solidification specimens, the ferrite remains relatively intact and only a small amount of the Sigma phase is present as secondary precipitates. Both the Chi phase and the Sigma phase are diffusion-controlled precipitates driven by Cr and Mo enrichment, and their formation requires a sufficiently long holding time at high temperatures [65,66,67]. Both DS 1 and DS 2 were found to contain a small amount of the Sigma phase; Specimen 2 (with a higher cooling rate) exhibited smaller precipitate sizes (about 10 μm) and fewer particles, indicating that increasing the cooling rate can suppress the nucleation and growth of precipitates. The cooling process of the continuously cast billet is more complex, resulting in more complete precipitate evolution. At the billet edge, with a cooling rate of 7.60 °C/s, both Sigma and Chi phases are formed in a coupled structure. At the quarter-thickness position, the cooling rate decreases to 0.70 °C/s, allowing more sufficient elemental diffusion, and the coupled precipitation of Sigma and Chi phases becomes more pronounced. At the center region, where the cooling rate is only 0.38 °C/s and the holding time is longest, part of the ferrite decomposes almost completely, and the Chi phase, as a precursor, is fully transformed into the stable Sigma phase, leaving only a single Sigma precipitate [50]. 

Comparing the results of this study with the precipitated phase characteristics of 316 stainless steels with similar Ni contents but different processes (such as electroslag remelting) reported in the literature, it is found that there are significant differences in the types, distribution, and evolution trends of the precipitated phases, as detailed below. In the study by Chu et al. [51] on precipitates in an 11.38% Ni 316L casting slab, the secondary precipitates consisted only of the Sigma phase, and the amount of the Sigma phase gradually increased from the edge, through the quarter-thickness, to the center. At the center, similar to the billet center in the present study, some ferrite had completely transformed into the Sigma phase. Similarly, Wang et al. [50], who studied a 12.2% Ni 316H electroslag remelted ingot with a Ni content close to that of the present billet, also investigated precipitates. They found that at the ingot edge, the secondary precipitates consisted only of the Chi phase, while at the ingot center, the secondary precipitates became Sigma and Chi phases, exhibiting a three-phase coupled structure (ferrite, Sigma, and Chi) similar to that observed at the edge and quarter-thickness positions of the present billet. From the above, it is clear that 316 stainless steels with different Ni contents and different casting processes exhibit considerable differences in ferrite morphology, ferrite content, and secondary precipitate characteristics.

### 4.4. The Influence of Thermal History on the Microstructure of As-Cast Billets and Directionally Solidified Specimens

Different thermal histories lead to distinct microstructures in materials [68,69]. A faster cooling rate may suppress diffusional transformation and retain nonequilibrium ferrite, whereas slower cooling can promote redistribution and transformation. Specifically, in the present study, the cooling rate of the continuous-casting billet decreases progressively from the edge to the center, resulting in ferrite morphologies evolving from fine granular and short rod-like at the edge, to skeletal at the quarter position, and finally to coarse clustered network and lath-like structures at the center. Owing to the prolonged high-temperature holding time at the center, some ferrite completely decomposes into the Sigma phase. In contrast, the directionally solidified specimens experience a short high-temperature holding time and insufficient elemental diffusion; their ferrite morphologies remain predominantly skeletal and the ferrite is retained more completely. This difference is mainly attributed to the more complex thermal history of the continuous-casting billet, which includes secondary cooling and air cooling stages. Meanwhile, directional solidification, by controlling the unidirectional heat flow direction, allows crystals to grow opposite to the heat flow, resulting in more regular grain arrangement and more uniform composition, with a relatively simpler thermal history; consequently, secondary precipitates are fewer and ferrite retention is more complete.

## 5. Conclusions and Future Work

### 5.1. Conclusions

(1) The residual ferrite morphologies of the 12.5% Ni 316L stainless steel billet and the directional solidification specimens differ significantly. In the billet, the ferrite at the edge, quarter-thickness, and center positions is granular and short rod-like, skeletal, and clustered network and lath-like, respectively. The two directional solidification specimens exhibit similar ferrite morphologies, both predominantly skeletal structures, with the main difference being that the ferrite in Directional Solidification Specimen 2 (higher withdrawal rate) is denser and finer.
(2) The ferrite contents at the billet edge, quarter-thickness, and center are 4.88%, 5.90%, and 8.99%, respectively; those of DS 1 and DS 2 are 6.4% and 7.7%, respectively. The billet shows a gradual increase in ferrite content from the edge to the center.
(3) EBSD results reveal a coupled structure of the secondary precipitated phases, i.e., Sigma and Chi phases, at the billet edge. At the quarter-thickness position, the coupled precipitation of Sigma and Chi phases becomes more pronounced. At the billet center, part of the ferrite decomposes completely, the Chi phase disappears, and only a single Sigma secondary precipitate remains. For the two directionally solidified samples, EBSD results reveal only a small amount of the Sigma phase, and the ferrite remains relatively intact.
(4) Thermo-Calc thermodynamic calculations for the solidification mode of 316L stainless steel yield an equilibrium solidification result of the AF mode, while the Scheil non-equilibrium solidification result yields the FA mode; the empirical formula based on chromium-nickel equivalent also predicts the solidification mode as the FA mode. Combining the ferrite morphology characteristics and the variation in ferrite content, it is ultimately determined that the solidification mode of the 12.5% Ni 316L stainless steel is the FA mode.
(5) Due to the differences in solidification processes at different positions of the billet, the ferrite morphology, content, and secondary precipitates vary significantly from the surface to the center. In contrast, directional solidification, by controlling the direction of unidirectional heat flow, the two directional solidification specimens exhibit similar ferrite morphology, fewer secondary precipitates, and more intact retained ferrite.

### 5.2. Future Work

This paper investigates the characteristics of residual ferrite and precipitated phases in two as-cast 12.5% nickel 316L stainless steels. The main focus in this study is the microstructure evolution. A limitation is that the billet and directionally solidified specimens are not entirely comparable in terms of thermal history and size. In addition, some aspects of the work may be explored in greater depth in the future. Regarding the possible macro-segregation in the continuous-casting billet, which can affect the phase distribution, particularly the ferrite content in the central region, the concentrations of C and N elements should also not be overlooked. Subsequent studies will consider macro-segregation as an important topic. Phase identification currently relies primarily on EBSD crystallographic calibration; in the future, more in-depth studies can be conducted using research methods such as TEM. The potential effects of δ-ferrite and precipitated phases on the corrosion resistance, toughness, ductility, and other properties of the material could also be considered as future work.

## Figures and Tables

**Table 1:** Composition of 316L austenitic stainless steel billet (mass percent, %).

C	Si	Mn	P	S	Cr	Ni	Mo	Cu	Al	Ti	N	Nb
0.014	0.50	1.06	0.031	0.0017	17.60	12.57	2.53	0.18	0.007	0.001	0.044	0.018

**Table 2:** Ferrite content, cooling rate, and phase precipitation of the 316L stainless steel billet and directional solidification specimens.

Sample Source	Sample	Ferrite Content (%)	Standard Deviation of Ferrite Content (%)	Secondary Dendrite Arm Spacing (μm)	Cooling Rate (°C/s)	Phase Constitution
Billet	Edge	4.88	1.025%	20.35	7.60	γ + δ + σ + χ
	One quarter	5.90	1.284%	50.39	0.70	γ + δ + σ + χ
	Center	8.99	1.928%	63.83	0.38	γ + δ + σ
Directional solidification	Sample 1	6.40	0.313%	32.78	2.17	γ + δ + σ
	Sample 2	7.70	0.464%	22.88	5.59	γ + δ + σ

## Data Availability

The original contributions presented in this study are included in the article. Further inquiries can be directed to the corresponding author.
